# Cationic Albumin Nanoparticles for Enhanced Drug Delivery to Treat Breast Cancer: Preparation and *In Vitro* Assessment

**DOI:** 10.1155/2012/686108

**Published:** 2011-12-08

**Authors:** Sana Abbasi, Arghya Paul, Wei Shao, Satya Prakash

**Affiliations:** ^1^Biomedical Technology and Cell Therapy Research Laboratory, Department of Biomedical Engineering, Faculty of Medicine, McGill University, 3775 University Street, Montreal, QC, Canada H3A 2B4; ^2^Artificial Cells and Organs Research Centre, Faculty of Medicine, McGill University, 3775 University Street, Montreal, QC, Canada H3A 2B4

## Abstract

Most anticancer drugs are greatly limited by the serious side effects that they cause. Doxorubicin (DOX) is an antineoplastic agent, commonly used against breast cancer. However, it may lead to irreversible cardiotoxicity, which could even result in congestive heart failure. In order to avoid these harmful side effects to the patients and to improve the therapeutic efficacy of doxorubicin, we developed DOX-loaded polyethylenimine- (PEI-) enhanced human serum albumin (HSA) nanoparticles. The formed nanoparticles were ~137 nm in size with a surface zeta potential of ~+15 mV, prepared using 20 **μ**g of PEI added per mg of HSA. Cytotoxicity was not observed with empty PEI-enhanced HSA nanoparticles, formed with low-molecular weight (25 kDa) PEI, indicating biocompatibility and safety of the nanoparticle formulation. Under optimized transfection conditions, approximately 80% of cells were transfected with HSA nanoparticles containing tetramethylrhodamine-conjugated bovine serum albumin. Conclusively, PEI-enhanced HSA nanoparticles show potential for developing into an effective carrier for anticancer drugs.

## 1. Introduction

Doxorubicin (Adriamycin) is a commonly used anti-cancer drug. It is most often used against breast and esophageal carcinomas, osteosarcoma and soft-tissue sarcomas, and Hodgkin's and non-Hodgkin's lymphomas [1]. The effectiveness of doxorubicin (DOX) in treating various types of cancers is greatly limited by the serious side effects caused by the drug. The initial side effects caused as a result of DOX administration include less serious symptoms, such as nausea, vomiting, myelosuppression, and arrhythmia, which are usually reversible [1]. However, DOX-associated cardiomyopathy and congestive heart failure have raised grave concern among health practitioners [2]. A widely researched approach of increasing the efficacy, while lowering the deleterious side effects caused by anti-cancer agents such as doxorubicin, is of developing nanoparticle-based drug delivery systems [3–5].

Various kinds of nanoparticles have been studied for the delivery of DOX, which include poly(butylcyanoacrylate) [6], poly(isohexylcyanoacrylate) [7], poly(lactic-co-glycolic acid [8], chitosan [9], gelatine [10], and liposomes [11]). In addition, Dreis et al. employed human serum albumin (HSA) nanoparticles of a size range between 150 and 500 nm to deliver DOX to a neuroblastoma cell line [3]. These nanoparticles showed a loading efficiency of 70–95% and an increased anti-cancer effect as compared to free DOX. The endogenous HSA serves as a suitable material for nanoparticle formation as albumin is naturally found in the blood and is thus easily degraded, nontoxic, and nonimmunogenic [12]. Albumin is an acidic protein and remains stable between pH range 4–9 and temperatures up to 60°C. In addition, clinical studies carried out with HSA particle formulations, Albunex [13] and Abraxane [14], have shown that albumin-based nanoparticles do not have any adverse effects on the body.

Furthermore, albumin-based nanoparticle delivery systems are easily accumulated in tumor tissue due to the enhanced permeability and retention (EPR) effect [15–17]. The vasculature in an active tumor is different from the vessels found in normal tissue. The distinctive tumor vasculature has the following properties: hypervasculature, poorly developed vascular architecture, a defective lymphatic drainage, and slow venous blood return [15, 16]. These characteristics lead to the preferential accumulation and retention of macromolecules and nanoparticles in the tumor tissue. Therefore, using a nanoparticle delivery system to deliver low-molecular-weight anti-cancer drugs will be passively targeted to the tumour tissue through the EPR effect [17]. In addition, studies have also suggested that accumulation of albumin-based nanoparticles within the tumor tissue is also because of transcytosis, which occurs by the binding of albumin to 60-kDa glycoprotein (gp60) receptor, which then results in the binding of gp60 with caveolin-1 and the consequent formation of transcytotic vesicles [12, 18]. Taking into consideration the factors mentioned above, HSA seems to be a suitable material to use for nanoparticle synthesis.

The surface properties of nanoparticles play a vital role in the cellular internalization of the particles. A neutrally charged surface does not show tendency of interacting with cell membranes, while charged groups found on nanoparticles are actively involved in nanomaterial-cell interaction [19]. Cho and Caruso found in their study of cellular internalization of gold nanoparticles that positively charged particles demonstrate greater adherence to the cell membrane and are thus taken up by the cells more than negatively and neutrally charged nanoparticles [20]. Cationic nanoparticles are shown to bind the negatively charged functional groups, such as sialic acid, found on cell surfaces and initiate translocation [19]. Due to the highly efficient transfection property of positively charged nanoparticles, many nanoparticle-based drug and gene delivery systems are positively charged. In this study, poly(ethylenimine) (PEI), a cationic polymer, has been used to coat the HSA nanoparticles in order to add stability and a positive surface charge to the nanoparticles. PEI may possess a linear or branched structure, with molecular weight ranging between 1 and 1000 kDa [21]. Typically, branched low-molecular-weight PEI (<25 kDa) has been observed to result in higher cellular uptake. As shown in our previous study, higher-molecular-weight PEI (70 kDa) leads to more cytotoxicity than lower-molecular-weight PEI (25 kDa) [22]. The most commonly used stabilizing agent for the preparation of HSA nanoparticles, glutaraldehyde, has been reported to interfere with the release of the encapsulated material [10, 23]. Thus, PEI is being employed as an alternative to glutaraldehyde in the current study.

PEI has been previously used to stabilize HSA nanoparticles. Initially, HSA nanoparticles stabilized using PEI were studied as vectors for protein delivery [24]. The osteoinductive growth factor, bone morphogenetic protein-2 (BMP-2), was encapsulated using PEI-coated albumin nanoparticles, and results showed that the bioactivity of the BMP-2 was retained, suggesting that the developed nanoparticles, are promising vectors for systemic protein administration [24]. In addition, Zhang et al. showed that the encapsulation efficiency of BMP-2 using PEI-coated albumin nanoparticles was >90% [25]. Furthermore, the efficacy of PEI-coated albumin nanoparticles for the delivery of BMP-2 was also confirmed *in vivo* with rats [26]. More recently, we showed that PEI-coated HSA nanoparticles were promising vectors for siRNA delivery [22].

**Figure 1:**
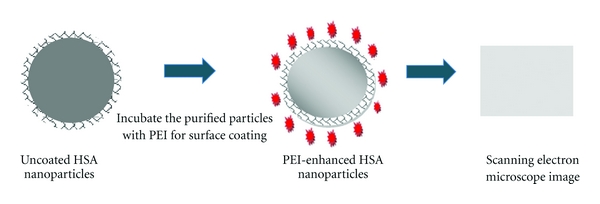
Formation of polyethylenimine- (PEI-) enhanced HSA nanoparticles.

In the current research study, the effectiveness of DOX-loaded polyethylenimine- (PEI-) enhanced HSA nanoparticles used against MCF-7 breast cancer cells was investigated. We prepared the nanoparticles using an ethanol desolvation method and characterized by measuring particle size, surface zeta potential, and cellular uptake [22, 27, 28]. The cytotoxicity of the developed DOX-loaded nanoparticles was assessed in comparison to free DOX at varying drug concentrations over different time points. Results were promising and suggest that the study needs to be followed up with an *in vivo* investigation of the DOX-loaded PEI-enhanced HSA nanoparticles (Figure 1).

## 2. Materials and Methods

### 2.1. Materials

Human serum albumin (HSA fraction V, purity 96–99%), 8% glutaraldehyde, and branched polyethylenimine (PEI) (M_*W*_ ~ 25,000) were purchased from Sigma Aldrich (ON, Canada). Doxorubicin hydrochloride was purchased from Calbiochem (Gibbstown, USA). All other reagents were purchased from Fischer (ON, Canada). Tetramethylrhodamine-conjugated bovine serum albumin (BSA) was purchased from Invitrogen (ON, Canada). To maintain the cell culture, the reagents such as fetal bovine serum, trypsin, Dulbecco's modified Eagle's Medium (DMEM), and Opti-MEM I Reduced Serum Medium were obtained from Invitrogen (ON, Canada). The breast cancer cell line, MCF-7, was purchased from ATCC (ON, Canada). Promega Cell-Titer 96 AQueous Non-Radioactive Cell Proliferation MTS Assay kit was purchased from Promega (Wis, USA).

### 2.2. Cell Culture

MCF-7 cells were cultured on tissue culture plates as per the manufacturer's instructions. MCF-7 cells were grown in Dulbecco's modified Eagle's Medium (Invitrogen) supplemented with 10% (v/v) fetal bovine serum (FBS) and placed in an incubator with 5% CO_2_ at 37°C. The cells used in the experiments were obtained from passages 5-6.

### 2.3. Preparation of DOX-Loaded PEI-Enhanced HSA Nanoparticles

PEI-coated HSA nanoparticles were prepared at room temperature using an ethanol desolvation technique [22, 27–29]. In brief, 20 mg of HSA was added to 1 mL of 10 mM NaCl (aq) under constant stirring (800 rpm) at room temperature. The solution was stirred for 10 min. After total dissolution, the solution was titrated to pH 8.5 with 1 N NaOH (aq) and stirred for 5 min. This aqueous phase was desolvated by the dropwise addition of ethanol to aqueous HSA solution under constant stirring. Ethanol was added until the HSA solution became turbid (~1-2 mL). Cross-linking agent, 8% glutaraldehyde, was added to form stable HSA particles. The obtained nanoparticles were centrifuged three times and washed with deionized water (dH_2_0), followed by resuspension in an equal volume of PBS. PEI dissolved in dH_2_0 was added to the nanoparticle preparation to allow PEI to form an outer coating due to electrostatic binding. For the preparation of drug-loaded HSA nanoparticles, doxorubicin was added to 1 mL HSA solution after pH adjustment and allowed to stir for 4 hrs, followed by ethanol addition. To determine the drug encapsulation efficiency, an indirect method was employed as shown by Sebak et al. [27]. The unloaded drug was quantified by measuring the free drug found in the supernatant of the prepared drug-loaded nanoparticles, using a UV spectrophotometer. Using the amount of unloaded drug, the drug-loaded quantity was determined (Total drug added (*μ*g)—free drug). The encapsulation efficiency was then calculated using the amount of drug loaded into the nanoparticles: amount of drug loaded (*μ*g)/theoretical maximum drug loading (*μ*g) [8].

### 2.4. Purification of PEI-Enhanced HSA Nanoparticles

PEI-coated HSA nanoparticles were ultracentrifuged (16500 g) for 12 min and added to 10 mM NaCl (aq) by vortexing and ultrasonication (Branson 2510). This method was repeated thrice to ensure complete removal of impurities.

### 2.5. Determining Particle Size and Surface Zeta Potential

The particle size and zeta potential were measured by electrophoretic laser Doppler anemometry, using a zeta potential analyzer (Brookhaven Instruments Corporation, USA). The nanoparticles were diluted 1 : 15 with distilled water prior to measurement [27].

### 2.6. Surface Characterization of PEI-Enhanced HSA Nanoparticles

The size and shape of the HSA nanoparticles were observed by transmission electron microscopy (TEM), using Philips CM200 200 kV TEM (Markham, Canada). The samples for TEM were prepared by ultracentrifuging the nanoparticles and washing with distilled water, followed by air drying the samples overnight to allow removal of moisture [22, 27, 29].

### 2.7. Transfection of MCF-7 Breast Cancer Cells with PEI-Enhanced HSA Nanoparticles

Prior to transfecting cells with nanoparticles, cells were washed with PBS and replenished with fresh serum-free DMEM. The PEI-coated HSA nanoparticles were prepared using 5% of Rhodamine-tagged HSA. The nanoparticles were purified and added to the cells. After 8 hrs of incubation of cells at 37°C with the nanoparticles, the culture medium was replaced with fresh DMEM, containing 10% FBS. Under the fluorescence microscope (TE2000-U, Nikon; USA), pictures were taken to assess the levels of transfection. The percentage of transfected cells was calculated by using the average of the number of cells exhibiting fluorescence under five different fields of view.

### 2.8. Cell Viability Assay

The number of surviving cells was assessed using the Promega Cell-Titer 96 AQueous Non-Radioactive Cell Proliferation MTS Assay kit. 3-(4,5-dimethylthiazol-2-yl)-5-(3-carboxymethoxyphenyl)-2-(4-sulfophenyl)-2H-tetrazolium, (MTS), and phenazine methosulfate reagents were used. Live cells reduce MTS to form formazan, a compound soluble in tissue-culture media. The amount of formazan is proportional to the number of living cells and can be quantified by measuring the absorbance of formazan, using 1420-040 Victor3 Multilabel Counter (Perkin Elmer, USA) at 490 nm. The intensity of the color produced by formazan indicates the viability of cells. MCF-7 cells were seeded onto a 96-well plate (10^4^ cells per well) 24 hrs before treatment. Cytotoxicity was measured at the predetermined time for each experiment using the MTS assay which was performed as per the manufacturer's protocol.

### 2.9. TUNEL Assay

The DeadEnd Colorimetric TUNEL System detects DNA fragmentation (an indicator of apoptosis) of each cell undergoing apoptosis. The fragmented ends of DNA are labelled by a modified TUNEL (TdT-mediated dUTP Nick-End Labeling) assay. The terminal deoxynucleotidyl transferase (TdT) enzyme adds a biotinylated nucleotide at the 3′-OH ends of DNA; the biotinylated nucleotides are conjugated with horseradish-peroxidase-labelled streptavidin. The peroxidase is then detected using its substrate, hydrogen peroxide, and the chromogen, diaminobenzidine (DAB). Following the manufacturer's protocol, the nuclei of apoptotic cells are stained brown.

## 3. Results and Discussion

### 3.1. Optimizing Coating of Cationic DOX-Loaded PEI-Enhanced HSA Nanoparticles

The desolvation technique used to prepare the HSA nanoparticles [22, 27, 30] is simple to perform; the synthesized particles were consistent in size, surface zeta potential, and morphology. The desolvation technique involves a liquid-liquid phase separation of an aqueous homogenous albumin solution, leading to the formation of a colloidal (or coacervate) phase that contains the nanoparticles [31]. In addition, the size of the nanoparticles formed by this technique can be altered based upon the various parameters of the technique, such as concentration and pH of HSA solution, volume and rate of ethanol addition [22, 29, 32]. In our previous research paper, we presented that the smallest nanoparticle size was achieved with 20 mg/mL HSA at pH 8.5 and ~1-2 mL of 100% ethanol [22]. These parameters were kept unchanged in this study as well. Glutaraldehyde cross-linking was carried out to stabilize the formed HSA nanoparticles before PEI surface coating; this also increases the drug entrapment ability of the HSA nanoparticles [3]. The encapsulation efficiency of DOX within PEI-enhanced HSA nanoparticles was calculated to be ~88.24 + 2.13%.

**Figure 2:**
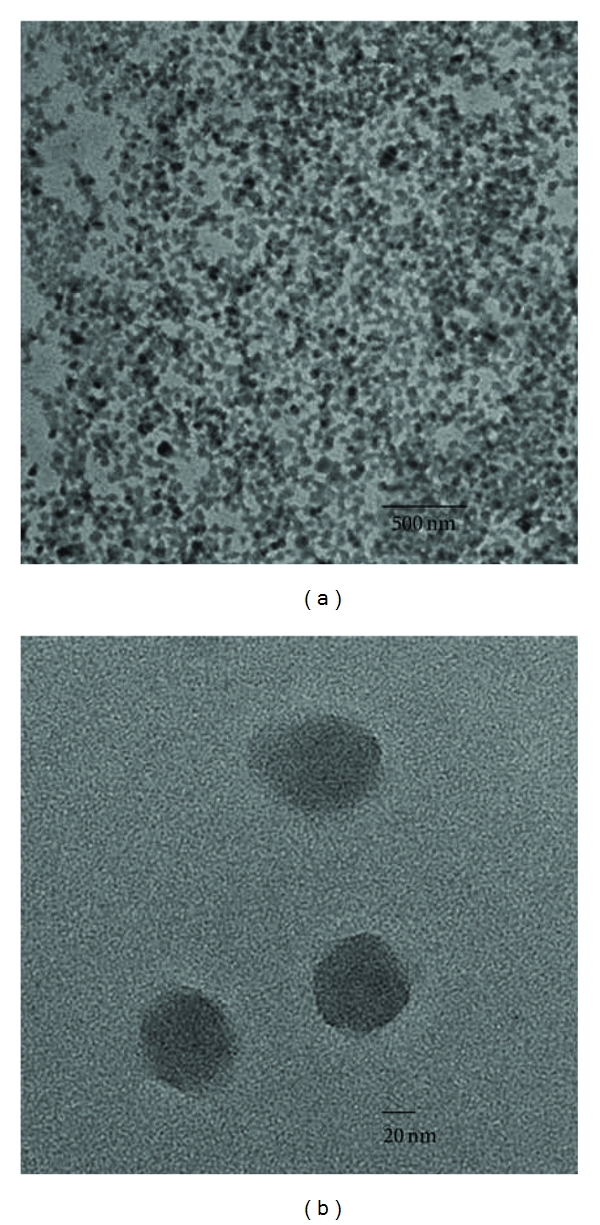
(a) Transmission electron microscope images of drug-loaded PEI-enhanced HSA nanoparticles. (b) Higher magnification image of the nanoparticles.

In the current study, PEI-enhanced HSA nanoparticles were prepared by coating the HSA nanoparticles that have a negative surface charge with electrostatic binding to the positively charged PEI. As HSA is an acidic protein, it carries a negative zeta potential in ~pH 8.5 and thus allows the positive PEI to bind to HSA nanoparticles [12, 33, 34]. The amount of PEI used for surface coating of the nanoparticles was optimized.  Table 1 shows that as the amount of PEI was increased, an increase in the particle size was observed, and the surface zeta potential became positive. This increase in size was gradual and could be attributed to the addition of the PEI surface coating or slight aggregation of the particles. The surface zeta potential increased from approximately −47 to +18 mV, clearly indicating that the PEI was successfully adsorbed to the nanoparticle surface. Furthermore, results presented in Table 2 show that 8 hrs of incubation at a stirring speed of 1000 rpm resulted in the smallest particle size and maximum zeta potential. Conditions were optimized to attain the smallest particle size and maximum zeta potential in order to achieve the highest cellular uptake [19]. Size dependence of cellular uptake has been studied previously [35]. For instance, Prabha et al. showed that smaller nanoparticles (~70 nm) experienced a 27-fold greater transfection than larger nanoparticles in COS-7 cell line, with all other parameters kept constant [35]. Similarly, surface charge of nanoparticles plays an important role in determining their transfection efficiency [19]. Harush-Frenkel et al. found that cationic nanoparticles resulted in rapid internalization through a clathrin-mediated pathway, while nanoparticles with a negative surface charge showed less efficient cellular uptake [36]. The TEM images shown in Figure 2 illustrate roughly spherical shape of the formed HSA nanoparticles of approximately 100 nm of size.

### 3.2. Increased Cellular Uptake of PEI-Enhanced HSA Nanoparticles

**Figure 3:**
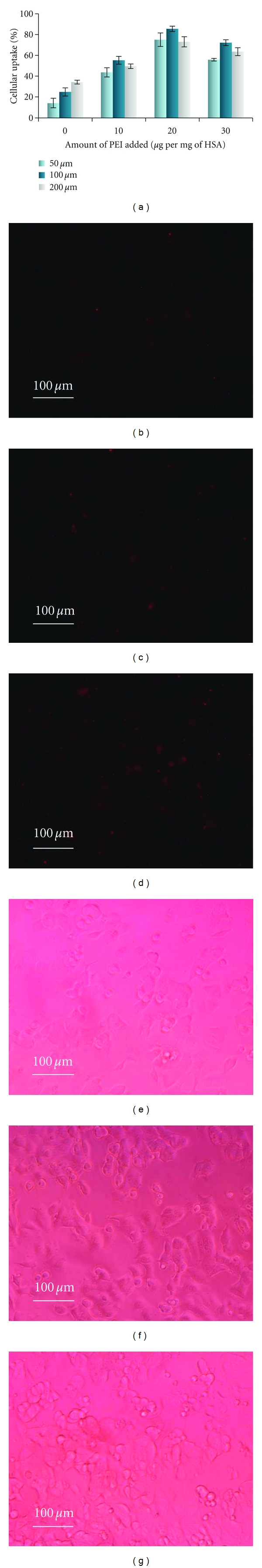
Cellular uptake of PEI-enhanced nanoparticles was assessed with respect to different amounts of PEI used for coating (mean ± S.D., *n* = 3). PEI-enhanced HSA nanoparticles were prepared using an ethanol desolvation technique with 20 mg/mL HSA. The nanoparticles were composed of 5% tetramethylrhodamine-conjugated BSA, and the cellular uptake was observed under a fluorescence microscope (TE2000-U, Nikon; USA). (a) Percentage of cellular uptake with nanoparticles prepared using 0, 10, 20, and 30 *μ*g of PEI per mg of HSA. Varying quantities of nanoparticle preparations were added to the cells: 50, 100, and 200 *μ*L. Fluorescence images of cellular uptake of different HSA nanoparticle preparations, consisting of tetramethylrhodamine-conjugated BSA, are shown; (b) uncoated HSA nanoparticles, (c) 10 *μ*g and (d) 30 *μ*g of PEI added per mg of HSA to form PEI-enhanced HSA nanoparticles. Corresponding bright field images are illustrated below (e, f, and g).

The cellular internalization of PEI-enhanced HSA nanoparticles was assessed by transfecting MCF-7 breast cancer cells with nanoparticles prepared with Rhodamine-tagged HSA. As shown in Figure 3, images were taken using a fluorescence microscope (TE2000-U, Nikon; USA). Cell transfection was measured with respect to the amount of PEI added to coat the nanoparticles. It is essential to optimize the amount of PEI used for coating the nanoparticles as this helps determine how much of the polymer is required to reach the maximum adsorption capacity of the surface of the nanoparticles and their corresponding surface zeta potential. Firstly, the lowest percentage of cell transfection was observed with uncoated nanoparticles, which can be attributed to the negative surface zeta potential of the uncoated HSA nanoparticles. Based on Figure 3(a), it can be concluded that increasing the amount of PEI, up to 20 *μ*g of PEI per mg of HSA, used for coating the nanoparticles leads to an increase in cell transfection. Further increasing the amount of PEI used for coating the nanoparticles did not translate into higher transfection efficiency. This observation could be explained by reaching the maximum capacity of PEI binding with the surface of HSA nanoparticles. Figures 3(b), 3(c), and 3(d) show corresponding fluorescence images of cellular uptake of PEI-enhanced HSA nanoparticles. The increase in cell transfection due to coating the nanoparticles with PEI is in agreement with previously published results. Cationic nanoparticles are shown to bind the negatively charged functional groups, such as sialic acid, found on cell surfaces and initiate transcytosis [19]. PEI-based nanoparticles have shown increased cellular uptake of siRNA. *In vivo* administration of siRNA delivered using PEI-based nanoparticles resulted in 80% decrease in the target gene expression; however, cytotoxicity was a concern [37, 38]. Therefore, a reasonable conclusion to draw from the results of the cell transfection experiment would be that the PEI adsorbed to the surface of the nanoparticles aids in the internalization of the particles. 

### 3.3. DOX Delivery with PEI-Enhanced HSA Nanoparticles to Kill Breast Cancer Cells

**Figure 4:**
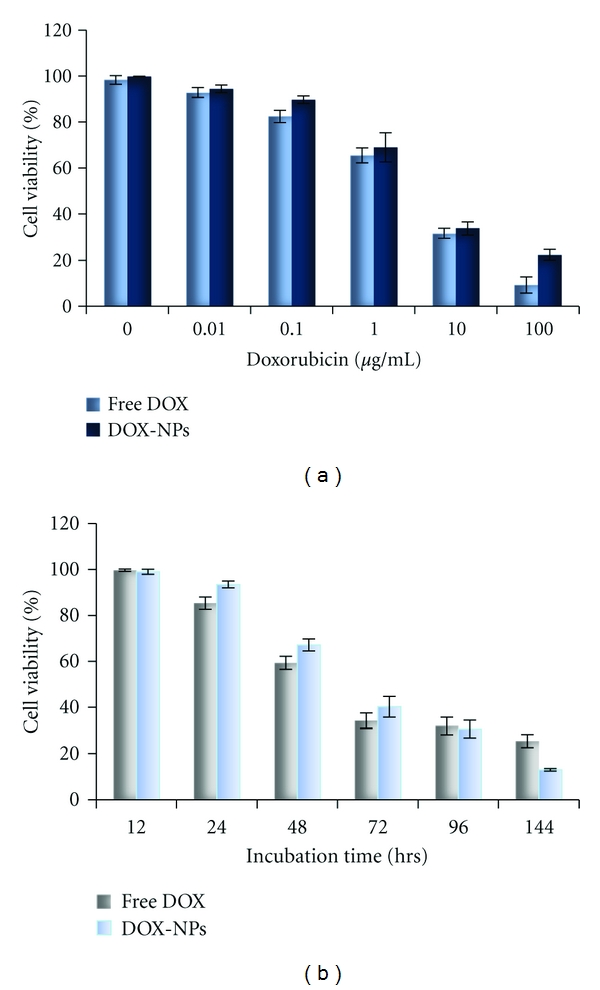
: (a) Dose-response cytotoxicity of DOX-loaded PEI-enhanced HSA nanoparticles as compared to free DOX administered to MCF-7 breast cancer cells in log-phase culture after 48 hrs of treatment with different concentrations of DOX. (b) Time of exposure: cytotoxicity resulting from DOX-loaded PEI-enhanced HSA nanoparticles versus free DOX over 96 hrs was measured. The concentration of DOX administered was 1 *μ*g/mL to MCF-7 breast cancer cells. Percentage of viable cells was assessed by an MTS assay and then compared to untreated cells in the control wells (mean ± S.D., *n* = 3).

**Figure 5:**
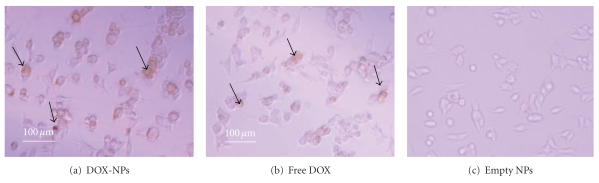
TUNEL assay to confirm cell death after DOX administration (24 hrs): (a) DOX-loaded PEI-enhanced HSA nanoparticles, (b) free DOX, and (c) empty PEI-enhanced HSA nanoparticles. The concentration of DOX administered was 1 *μ*g/mL to MCF-7 breast cancer cells grown in a 96-well plate. The black arrows point towards cells showing TUNEL staining.

The efficacy of anti-cancer chemotherapy is limited by the cytotoxic effect on healthy cells due to a lack of selectivity of the drugs and poor uptake of the therapeutics by the tumor cells [19, 39, 40]. Doxorubicin, a strong antineoplastic agent, has been shown to cause irreversible cardiomyopathy, which could also lead to congestive heart failure [1, 19, 40]. In order to overcome this issue, many researchers have tried delivering DOX by nanoparticles that reduce the amount of drug reaching cardiac tissue while increasing the accumulation of the drug-loaded nanoparticles in the tumor tissue [7, 9, 32, 41–43]. Furthermore, by incorporating a layer of PEI on the surface of the HSA nanoparticles, we aimed to increase their cellular uptake in the tumor tissue. Previously, uncoated HSA nanoparticles were studied for the delivery of DOX to neuroblastoma cell lines. Results suggested that DOX delivered using nanoparticles was more cytotoxic against cancer cells as compared to free DOX. In our study, we observed that the cytotoxicity of DOX-loaded nanoparticle and free DOX against MCF-7 breast cancer cells was about the same after 48 hrs as the DOX concentration was increased, shown in Figure 4(a). However, assessing the cytotoxicity at different time points in Figure 4(b) showed that DOX-loaded nanoparticles led to a greater decrease in cell viability as compared to free DOX after 144 hrs. This observation can be explained by the slow release of DOX from the nanoparticles. These results would be more effective *in vivo* as the free drug would diffuse out of the tumor tissue, while the nanoparticles would accumulate within the tumor tissue due to the EPR effect and release the drug over time. Images of treated cells after TUNEL staining in Figures  5(a), 5(b), and 5(c) confirm that the cytotoxic effect of DOX-loaded nanoparticles was comparable to free DOX. Figure  5(c) shows that the cells remained healthy and viable after the addition of PEI-enhanced HSA nanoparticles, suggesting that the nanoparticle formulation does not have cytotoxic effects.

## 4. Conclusion

In our current study, we used modified HSA nanoparticles by adding an outer coating of the polyethylenimine (PEI) to improve the therapeutic index of doxorubicin against MCF-7 breast cancer cells. The nanoparticles prepared were characterized based upon size and surface charge with respect to the amount of PEI used for coating. A rise in the surface zeta potential of the nanoparticles confirms the electrostatic binding of PEI with the surface of HSA nanoparticles. Different microscopic techniques were employed to observe the shape, dispersion, and morphology of the nanoparticles. PEI-enhanced HSA nanoparticles resulted in a higher cell transfection percentage, indicating that the addition of the layer of cationic polymer did improve cell penetration of the particles. PEI-enhanced HSA nanoparticles illustrated a more potent cytotoxic effect on MCF-7 breast cancer cells over longer time duration. The results shown in this study are promising and set a platform for further examining the suitability of this PEI-enhanced delivery system *in vivo*. 

## Figures and Tables

**Table 1:** Effect of the amount of PEI added (*μ*g per mg of HSA) on the physical characteristics of drug-loaded PEI-enhanced HSA nanoparticles prepared at pH 8.5, 20 mg/mL HSA (mean ± S.D., *n* = 3).

Amount of PEI (*μ*g) added per mg of HSA	Particle size (nm)	Zeta potential (mV)
0	99.63 ± 6.01	−46.9 ± 5.06
10	105.6 ± 8.07	+6.14 ± 1.11
20	121.7 ± 2.78	+12.3 ± 0.18
30	137.2 ± 8.20	+17.92 ± 1.04
40	135.5 ± 4.27	+18.38 ± 3.7

**Table 2:** Effect of incubation time for PEI coating and stirring speed during the desolvation step on the physical characteristics of drug-loaded PEI-enhanced HSA nanoparticles, prepared with 20 mg/mL HSA and 30 *μ*g of PEI added per mg of HSA (mean ± S.D., *n* = 3).

Time of incubation with PEI (hrs)	Stirring speed (rpm)	Particle size (nm)	Zeta potential (mV)
4	250	412.76 ± 12.7	8.94 ± 0.12
	500	248.43 ± 1.7	7.20 ± 0.19
	1000	130.47 ± 11.3	4.24 ± 0.08

8	250	362.77 ± 0.65	17.4 ± 0.36
	500	218.57 ± 15.9	19.14 ± 0.51
	1000	100.73 ± 3.93	18.39 ± 0.27

12	250	332.67 ± 16.2	16.13 ± 0.91
	500	205.17 ± 8.16	10.99 ± 0.71
	1000	111.53 ± 4.72	13.73 ± 0.36

## References

[1] Singal PK, Iliskovic N (1998). Doxorubicin-induced cardiomyopathy. *The New England Journal of Medicine*.

[2] Singal PK, Li T, Kumar D, Danelisen I, Iliskovic N (2000). Adriamycin-induced heart failure: mechanism and modulation. *Molecular and Cellular Biochemistry*.

[3] Dreis S, Rothweiler F, Michaelis M, Cinatl J, Kreuter J, Langer K (2007). Preparation, characterisation and maintenance of drug efficacy of doxorubicin-loaded human serum albumin (HSA) nanoparticles. *International Journal of Pharmaceutics*.

[4] Hans ML, Lowman AM (2002). Biodegradable nanoparticles for drug delivery and targeting. *Current Opinion in Solid State and Materials Science*.

[5] Seigneuric R, Markey L, Nuyten DSA (2010). From nanotechnology to nanomedicine: applications to cancer research. *Current Molecular Medicine*.

[6] Gulyaev AE, Gelperina SE, Skidan IN, Antropov AS, Kivman GY, Kreuter J (1999). Significant transport of doxorubicin into the brain with polysorbate 80-coated nanoparticles. *Pharmaceutical Research*.

[7] Cuvier C, Roblot-Treupel L, Millot JM (1992). Doxorubicin-loaded nanospheres bypass tumor cell multidrug resistance. *Biochemical Pharmacology*.

[8] Park J, Fong PM, Lu J (2009). PEGylated PLGA nanoparticles for the improved delivery of doxorubicin. *Nanomedicine: Nanotechnology, Biology, and Medicine*.

[9] Janes KA, Fresneau MP, Marazuela A, Fabra A, Alonso MJ (2001). Chitosan nanoparticles as delivery systems for doxorubicin. *Journal of Controlled Release*.

[10] Leo E, Vandelli MA, Cameroni R, Forni F (1997). Doxorubicin-loaded gelatin nanoparticles stabilized by glutaraldehyde: involvement of the drug in the cross-linking process. *International Journal of Pharmaceutics*.

[11] Lukyanov AN, Elbayoumi TA, Chakilam AR, Torchilin VP (2004). Tumor-targeted liposomes: doxorubicin-loaded long-circulating liposomes modified with anti-cancer antibody. *Journal of Controlled Release*.

[12] Kratz F (2008). Albumin as a drug carrier: design of prodrugs, drug conjugates and nanoparticles. *Journal of Controlled Release*.

[13] Feinstein SB, Cheirif J, Ten Cate FJ (1990). Safety and efficacy of a new transpulmonary ultrasound contrast agent: initial multicenter clinical results. *Journal of the American College of Cardiology*.

[14] Ibrahim NK, Desai N, Legha S (2002). Phase I and pharmacokinetic study of ABI-007, a Cremophor-free, protein-stabilized, nanoparticle formulation of paclitaxel. *Clinical Cancer Research*.

[15] Maeda H, Wu J, Sawa T, Matsumura Y, Hori K (2000). Tumor vascular permeability and the EPR effect in macromolecular therapeutics: a review. *Journal of Controlled Release*.

[16] Maeda H (2010). Tumor-selective delivery of macromolecular drugs via the EPR effect: background and future prospects. *Bioconjugate Chemistry*.

[17] Son YJ, Jang JS, Cho YW (2003). Biodistribution and anti-tumor efficacy of doxorubicin loaded glycol-chitosan nanoaggregates by EPR effect. *Journal of Controlled Release*.

[18] Desai N, Trieu V, Yao Z (2006). Increased antitumor activity, intratumor paclitaxel concentrations, and endothelial cell transport of cremophor-free, albumin-bound paclitaxel, ABI-007, compared with cremophor-based paclitaxel. *Clinical Cancer Research*.

[19] Verma A, Stellacci F (2010). Effect of surface properties on nanoparticle-cell interactions. *Small*.

[20] Cho J, Caruso F (2005). Investigation of the interactions between ligand-stabilized gold nanoparticles and polyelectrolyte multilayer films. *Chemistry of Materials*.

[21] Kichler A (2004). Gene transfer with modified polyethylenimines. *Journal of Gene Medicine*.

[22] Abbasi S, Paul A, Prakash S Investigation of siRNA-loaded polyethylenimine-coated human serum albumin nanoparticle complexes for the treatment of breast cancer.

[23] Segura S, Espuelas S, Renedo MJ, Irache JM (2005). Potential of albumin nanoparticles as carriers for interferon gamma. *Drug Development and Industrial Pharmacy*.

[24] Wang G, Siggers K, Zhang S (2008). Preparation of BMP-2 containing bovine serum albumin (BSA) nanoparticles stabilized by polymer coating. *Pharmaceutical Research*.

[25] Zhang S, Wang G, Lin X (2008). Polyethylenimine-coated albumin nanoparticles for BMP-2 delivery. *Biotechnology Progress*.

[26] Zhang S, Doschak MR, Uludağ H (2009). Pharmacokinetics and bone formation by BMP-2 entrapped in polyethylenimine-coated albumin nanoparticles. *Biomaterials*.

[27] Sebak S, Mirzaei M, Malhotra M, Kulamarva A, Prakash S (2010). Human serum albumin nanoparticles as an efficient noscapine drug delivery system for potential use in breast cancer: preparation and in vitro analysis. *International Journal of Nanomedicine*.

[28] Weber C, Kreuter J, Langer K (2000). Desolvation process and surface characteristics of HSA-nanoparticles. *International Journal of Pharmaceutics*.

[29] Langer K, Balthasar S, Vogel V, Dinauer N, Von Briesen H, Schubert D (2003). Optimization of the preparation process for human serum albumin (HSA) nanoparticles. *International Journal of Pharmaceutics*.

[30] Khan A, Paul A, Abbasi S, Prakash S (2011). Mitotic and antiapoptotic effects of nanoparticles coencapsulating human VEGF and human angiopoietin 1 on vascular endothelial cells. *International Jounral of Nanomedicine*.

[31] Menger FM, Sykes BM (1998). Anatomy of a coacervate. *Langmuir*.

[32] Lin W, Coombes AG, Davies MC, Davis SS, Illum L (1993). Preparation of sub-100 nm human serum albumin nanospheres using a pH-coacervation method. *Journal of Drug Targeting*.

[33] Singh HD, Wang G, Uludağ H, Unsworth LD (2010). Poly-L-lysine-coated albumin nanoparticles: stability, mechanism for increasing in vitro enzymatic resilience, and siRNA release characteristics. *Acta Biomaterialia*.

[34] Torchilin VP, Rammohan R, Weissig V, Levchenko TS (2001). TAT peptide on the surface of liposomes affords their efficient intracellular delivery even at low temperature and in the presence of metabolic inhibitors. *Proceedings of the National Academy of Sciences of the United States of America*.

[35] Prabha S, Zhou WZ, Panyam J, Labhasetwar V (2002). Size-dependency of nanoparticle-mediated gene transfection: studies with fractionated nanoparticles. *International Journal of Pharmaceutics*.

[36] Harush-Frenkel O, Rozentur E, Benita S, Altschuler Y (2008). Surface charge of nanoparticles determines their endocytic and transcytotic pathway in polarized MDCK cells. *Biomacromolecules*.

[37] Hunter AC (2006). Molecular hurdles in polyfectin design and mechanistic background to polycation induced cytotoxicity. *Advanced Drug Delivery Reviews*.

[38] Tietze N, Pelisek J, Philipp A (2008). Induction of apoptosis in murine neuroblastoma by systemic delivery of transferrin-shielded siRNA polyplexes for downregulation of Ran. *Oligonucleotides*.

[39] Maughan KL, Lutterbie MA, Ham PS (2010). Treatment of breast cancer. *American Family Physician*.

[40] Partridge AH, Burstein HJ, Winer EP (2001). Side effects of chemotherapy and combined chemohormonal therapy in women with early-stage breast cancer. *Journal of the National Cancer Institute. Monographs*.

[41] Bennis S, Chapey C, Couvreur P, Robert J (1994). Enhanced cytotoxicity of doxorubicin encapsulated in polyisohexylcyanoacrylate nanospheres against multidrug-resistant tumour cells in culture. *European Journal of Cancer Part A*.

[42] Brannon-Peppas L, Blanchette JO (2004). Nanoparticle and targeted systems for cancer therapy. *Advanced Drug Delivery Reviews*.

[43] Dhankhar R, Vyas SP, Jain AK, Arora S, Rath G, Goyal AK (2010). Advances in novel drug delivery strategies for breast cancer therapy. *Artificial Cells, Blood Substitutes, and Biotechnology*.

